# Supplementary material for: Competition between transmission lineages mediated by human mobility shapes seasonal influenza epidemics in the US
Source: Nat Commun. 2025 May 17;16:4605. doi: 10.1038/s41467-025-59757-4 (PMC12085627; doi:10.1038/s41467-025-59757-4)
Supplement: Supplementary file 1 — Supplementary Information [file 41467_2025_59757_MOESM1_ESM.pdf]

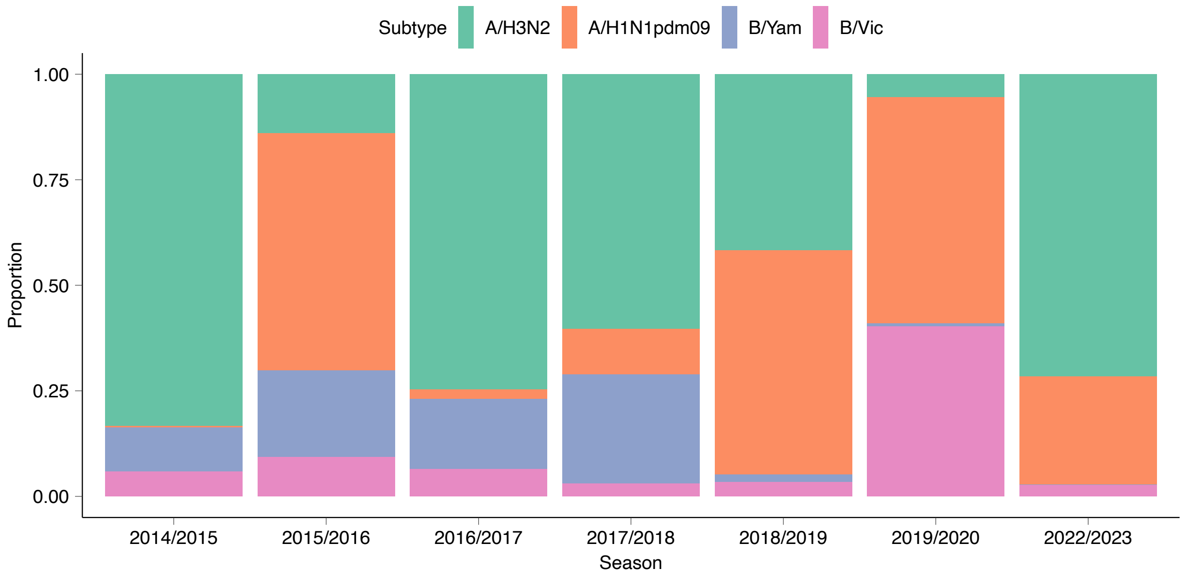

Supplementary Fig. 1. Nation-wide epidemic compositions for the seasons included in the analysis, as computed from virological surveillance data.

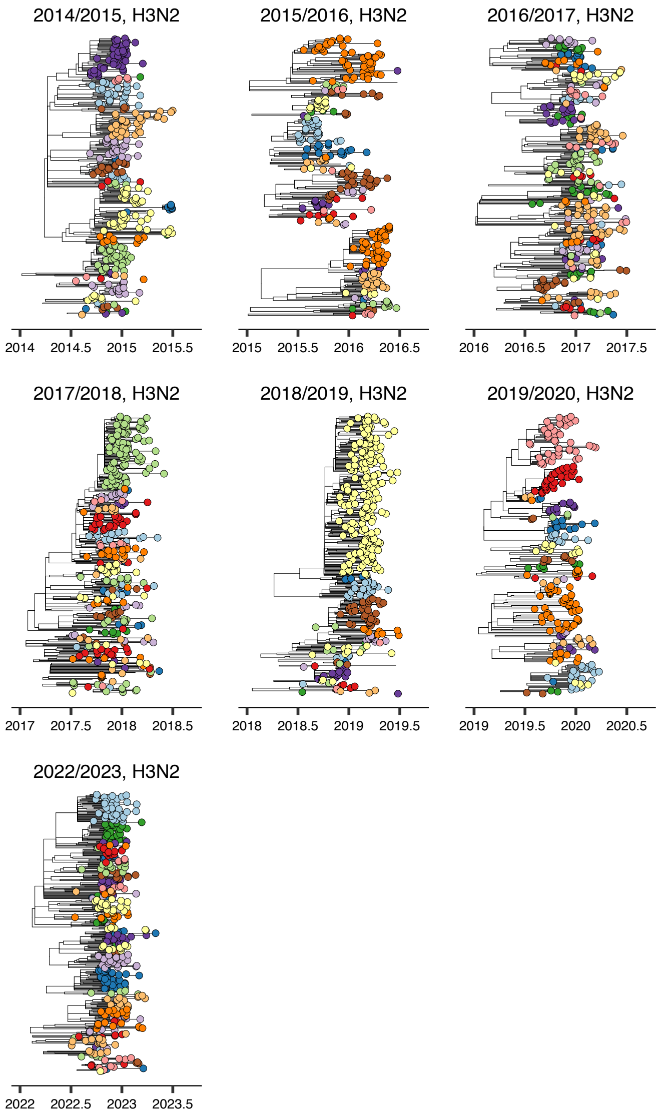

Supplementary Fig. 2. Phylogenies for the A/H3N2 subtype, colored by transmission lineage.

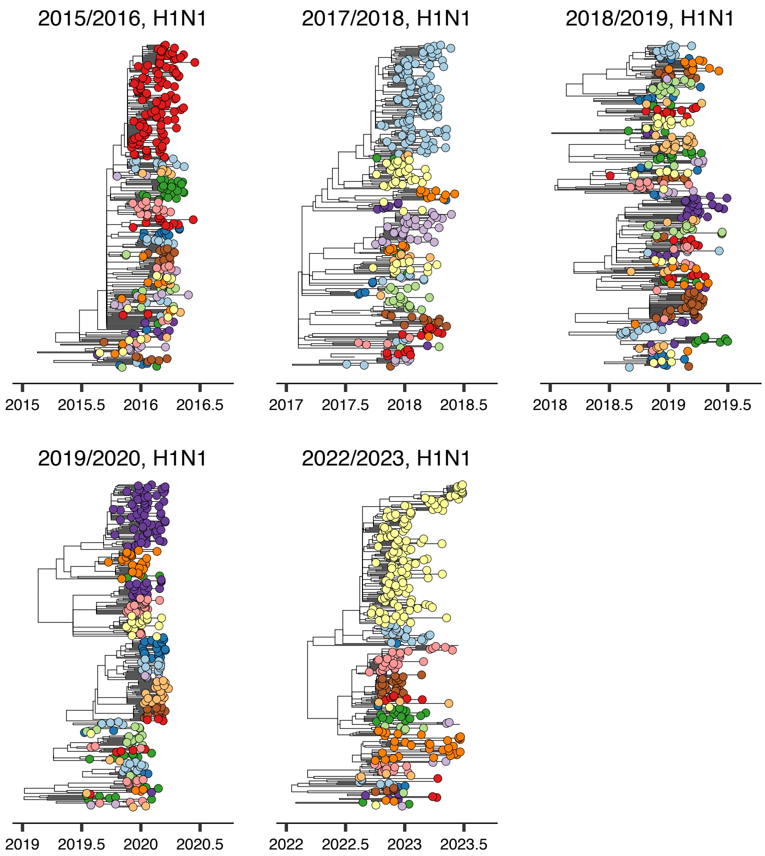

Supplementary Fig. 3. Phylogenies for the A/H1N1pdm09 subtype, colored by transmission lineage.

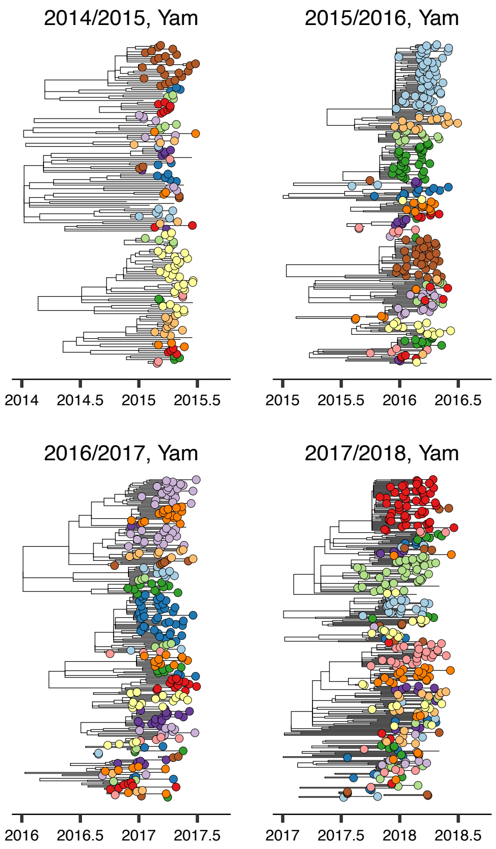

Supplementary Fig. 4. Phylogenies for the B/Yamagata lineage, colored by transmission lineage.

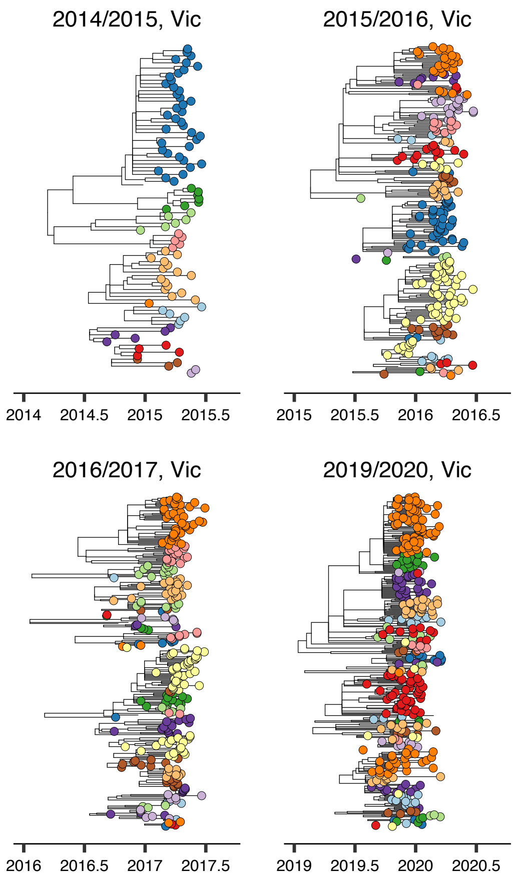

Supplementary Fig. 5. Phylogenies for the B/Victoria lineage, colored by transmission lineage.

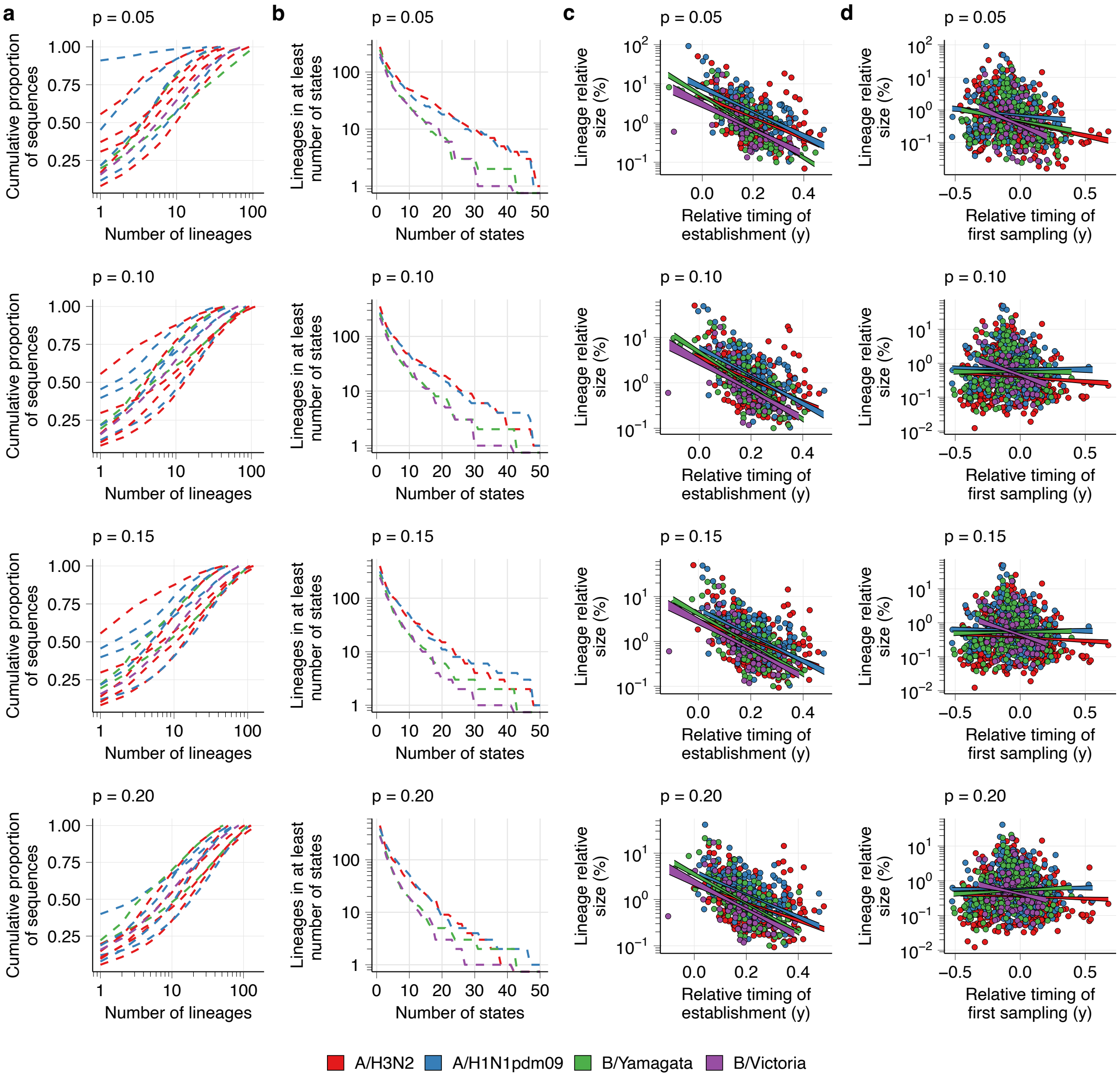

Supplementary Fig. 6. Analyses of seasonal influenza virus epidemic transmission lineage structure, for different values for *p*, the proportion of coalescent events that must occur within a month after the putative transmission lineage's root in the clustering procedure. **a** Lineage size distribution by season and subtype. Each line represents the cumulative proportion of sequences that is accounted for by a number of lineages on the *x*-axis. **b** The number of subtype-specific lineages that accounted for >5% of sequences in a season-subtype in at least the number of states on the *x*-axis. **c** Relationship between the first collection date of virus in a lineage and the lineage's country-wide size normalized by state. Lineage sampling dates were computed relative to the timing of nation-wide epidemic onset, which was defined as the first week in which >5% of the season's cumulative positive tests had been collected. Lines correspond to 50% CI given linear fit. **d** Relationship between the timing of establishment of substantial circulation of a lineage and its country-wide size. Lineage establishment timing was computed relative to nation-wide epidemic onset analogous to **d**.

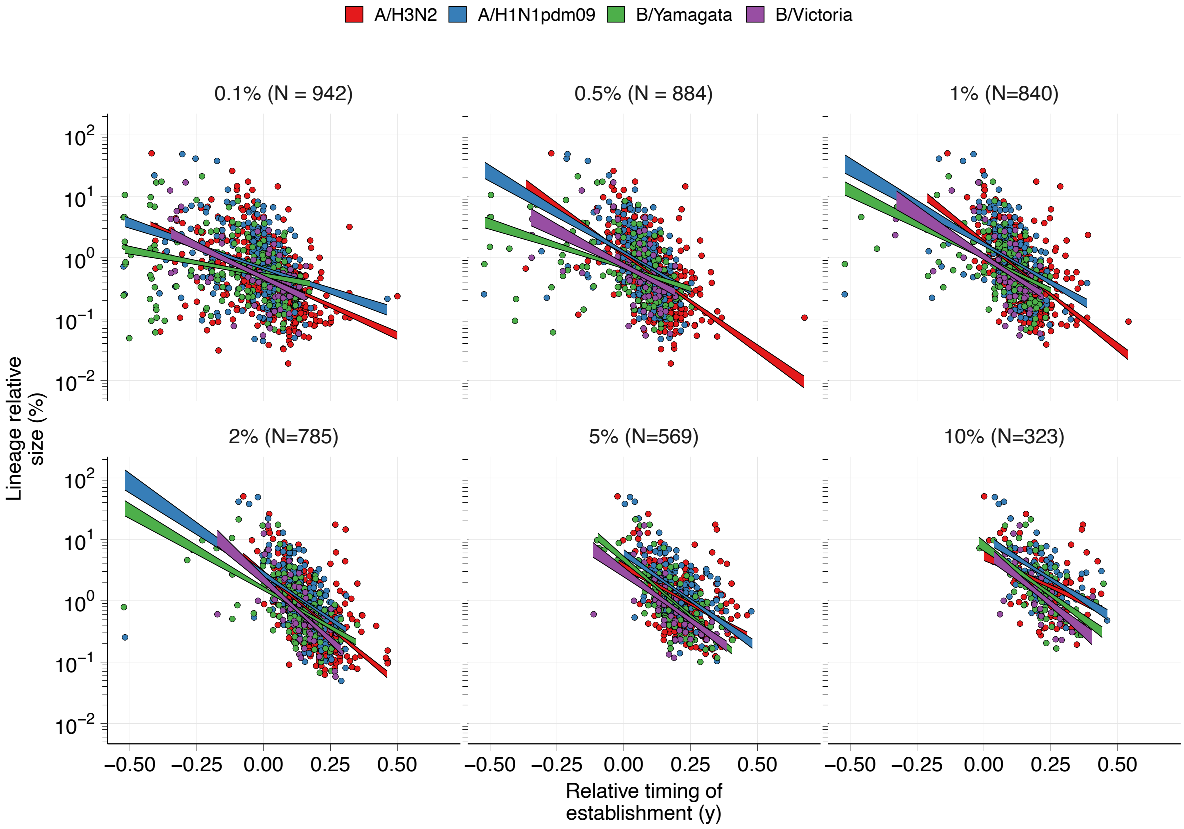

Supplementary Fig. 7. Correlation between relative lineage establishment timing and lineage nationwide relative size, for different cumulative incidence thresholds used to determine establishment timing. Each panel represents a different threshold, with *N* representing the number of lineages for that threshold. Lines correspond to 50% CI given linear fit.

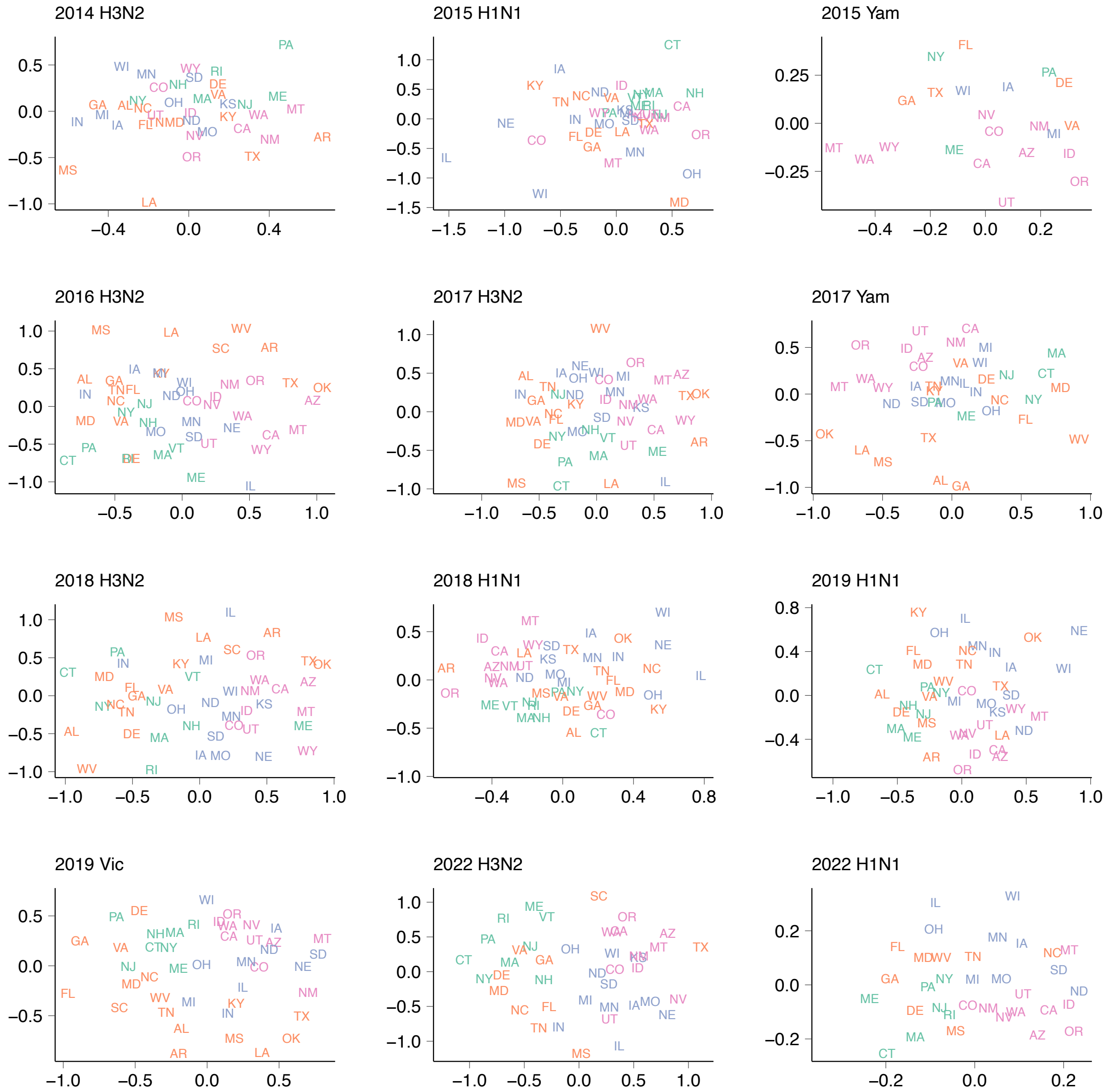

Supplementary Fig. 8. Multi-dimensional scaling plot of the pairwise lineage composition Bray-Curtis similarity among states by season and subtype, for each of the season-subtype combinations that accounted for >20% of detections in the respective season. States are colored by census region (green: Northeast; orange: South; blue: Midwest; pink: West).

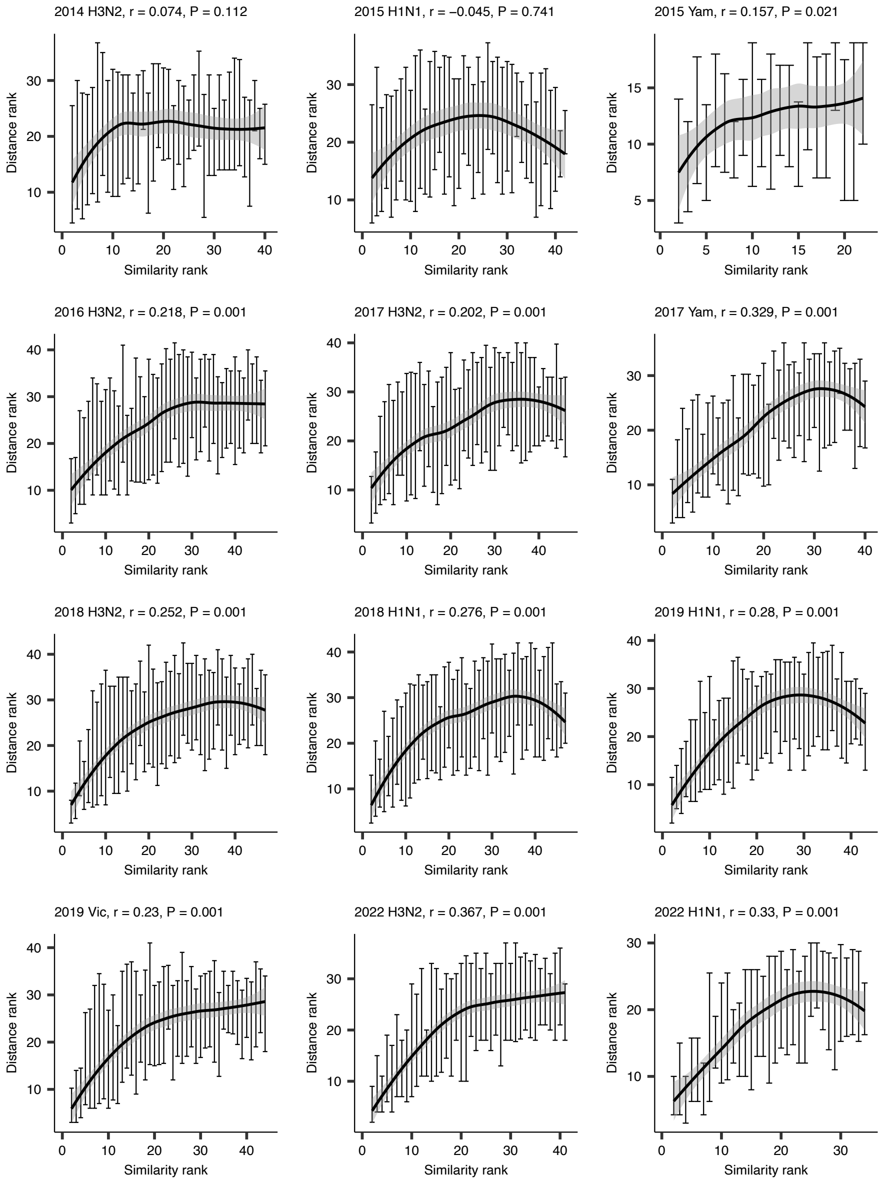

Supplementary Fig. 9. Relationship between pairwise transmission lineage compositional similarity and pairwise centroid distance rank. Vertical lines show 50% CI for each value of rank similarity, line corresponds to LOESS fit to medians. Each panel corresponds to a different subtype and season. *r* and *P* values are for a Mantel test for compositional similarity and centroid distance.

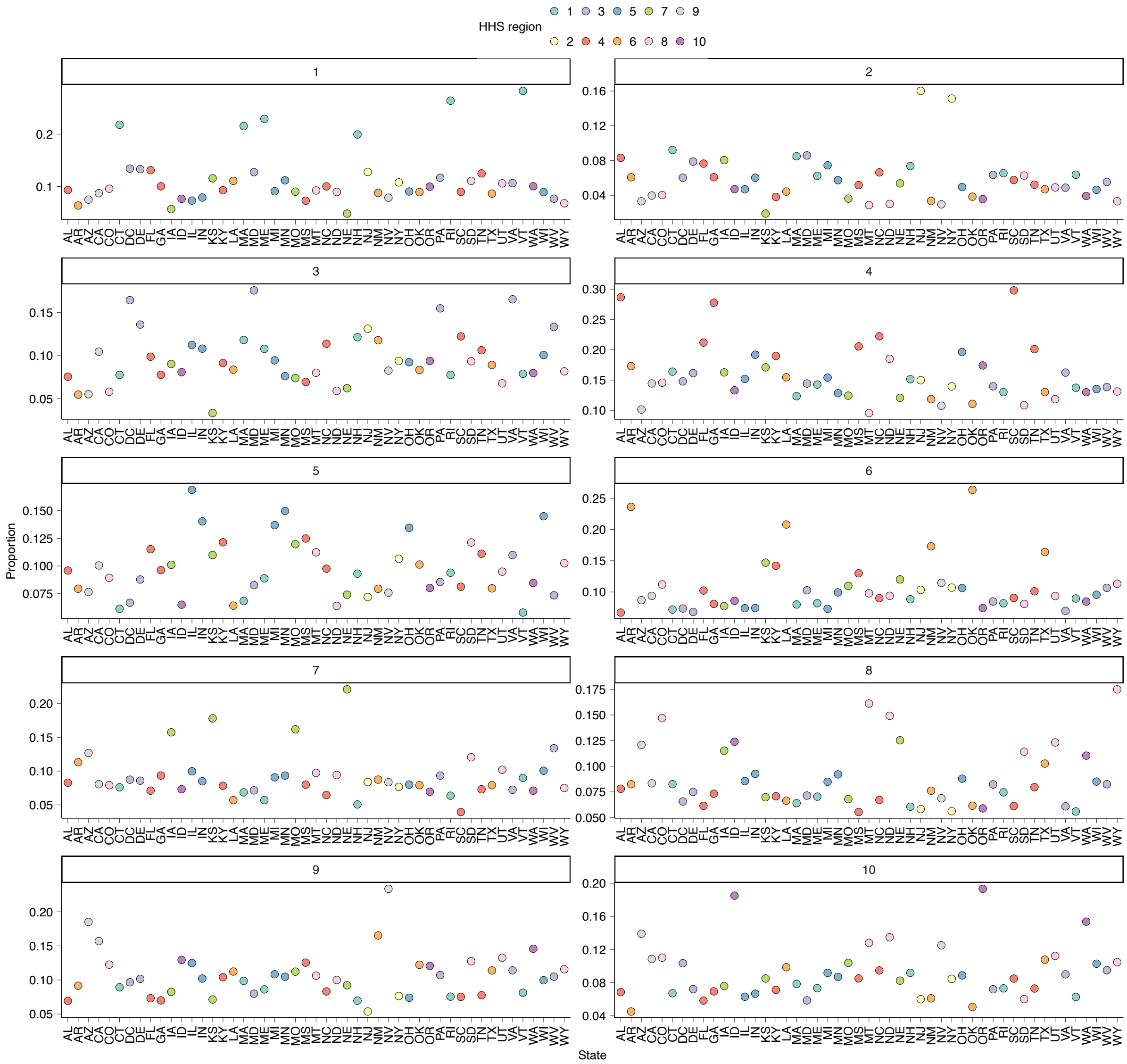

Supplementary Fig. 10. The proportion (*y*-axis) of sequences in each state (*x*-axis) that can be attributed to lineages that originally expanded from each HHS region. Each subpanel corresponds to a source HHS region. Results are for the subsampling strategy with uniform sampling across HHS regions.

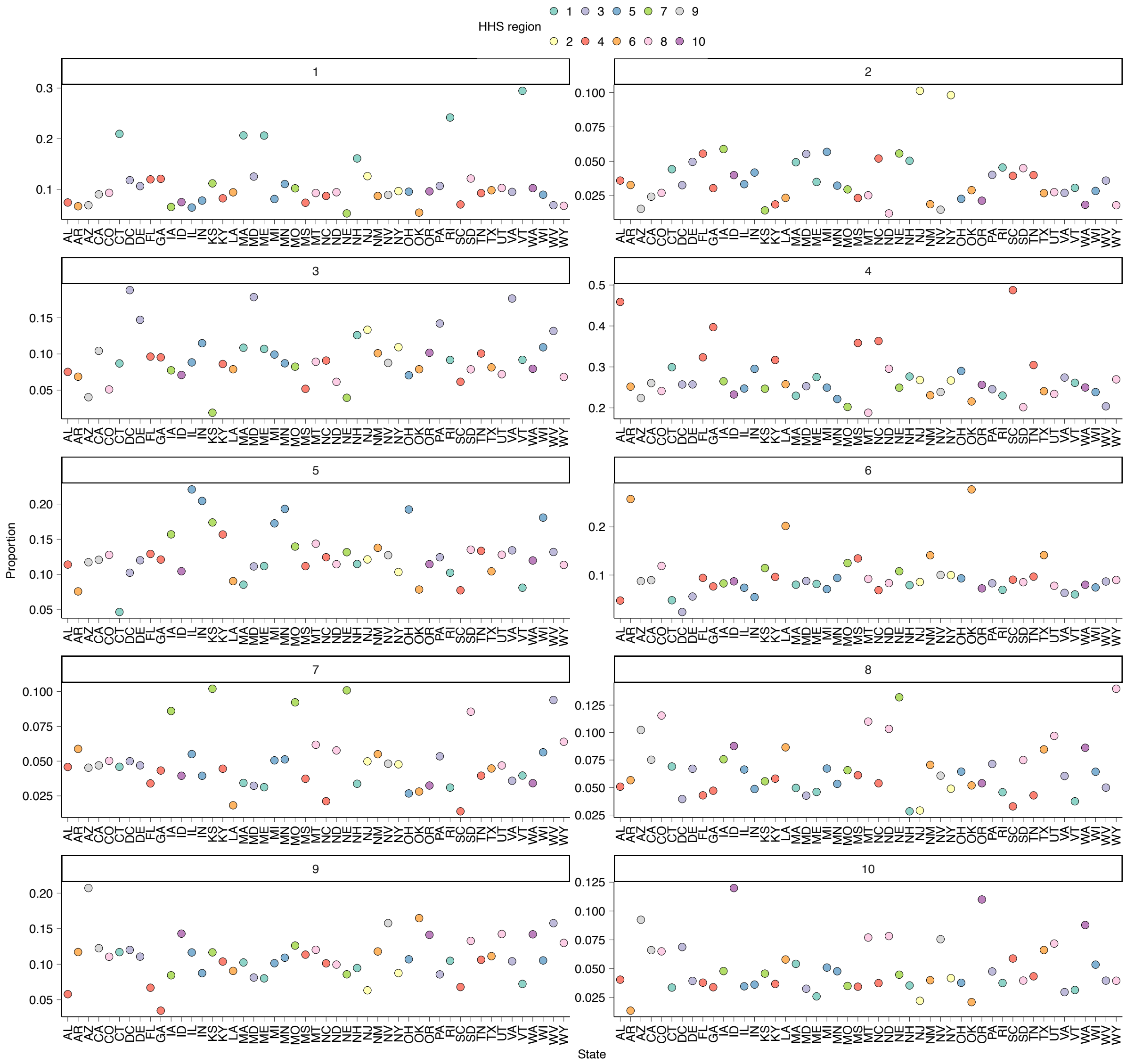

Supplementary Fig. 11. The proportion (*y*-axis) of sequences in each state (*x*-axis) that can be attributed to lineages that originally expanded from each HHS region. Each subpanel corresponds to a source HHS region. Results are for the subsampling strategy with population size-based sampling across HHS regions.

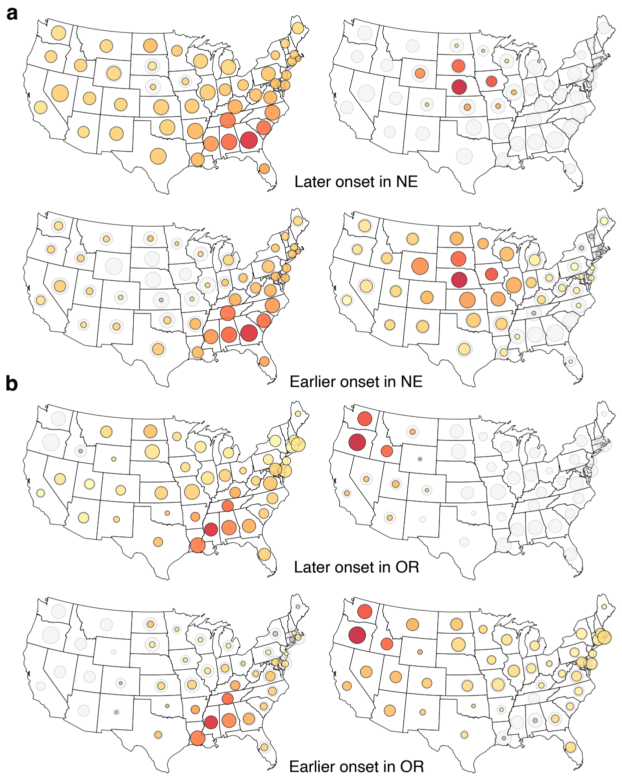

Supplementary Fig. 12. The simulated spread of the two largest lineages in the 2018/2019 A/H3N2 season (**a**) and 2017/2018 A/H1N1pdm09 season (**b**), had either lineage established four weeks sooner or four weeks later. Light grey circles represent the total proportion of sequences in that state that are accounted for by the lineages that were simulated, to account for the fact that simulations only incorporated a subset of all lineages; circles for the simulated lineages have their size scaled such that the sum of simulated lineages' sizes for each state is proportional to the proportion of sequences accounted for by the simulated lineages in that state (i.e., the light grey area).

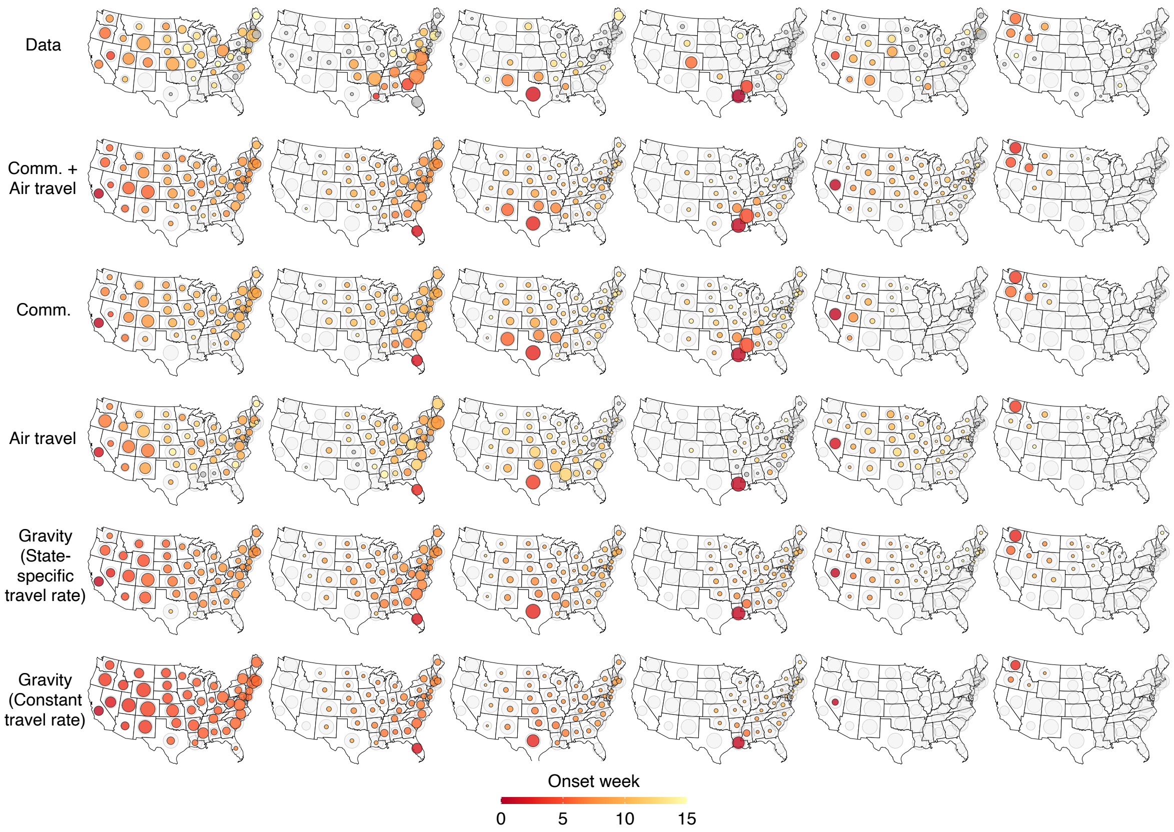

Supplementary Fig. 13. Top row of maps represents the reconstructed spread and distribution of each of the six largest lineages in the 2019/2020 B/Victoria season. Other rows correspond to simulations using different parameterizations of human mobility: a combination of commuting and air travel data, commuting only, air travel only, a gravity model with a state-specific rate of outward travel, and a gravity model with a rate of outward travel that is uniform across states, respectively. Circle sizes are scaled as in Fig. 3.

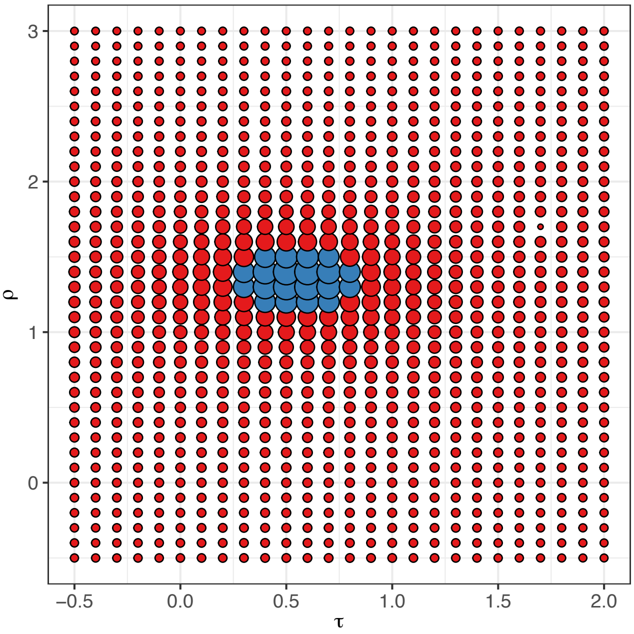

Supplementary Fig. 14. Model fit for a singly-constrained gravity model fit to the 2019/2020 B/Victoria season. Point size is proportional to the likelihood of each simulation for corresponding distance parameter ρ and destination population size parameter τ. Blue color corresponds to parameter sets included in the 95% CI calculated using profile likelihoods; red color corresponds to parameter sets outside the 95% CI.

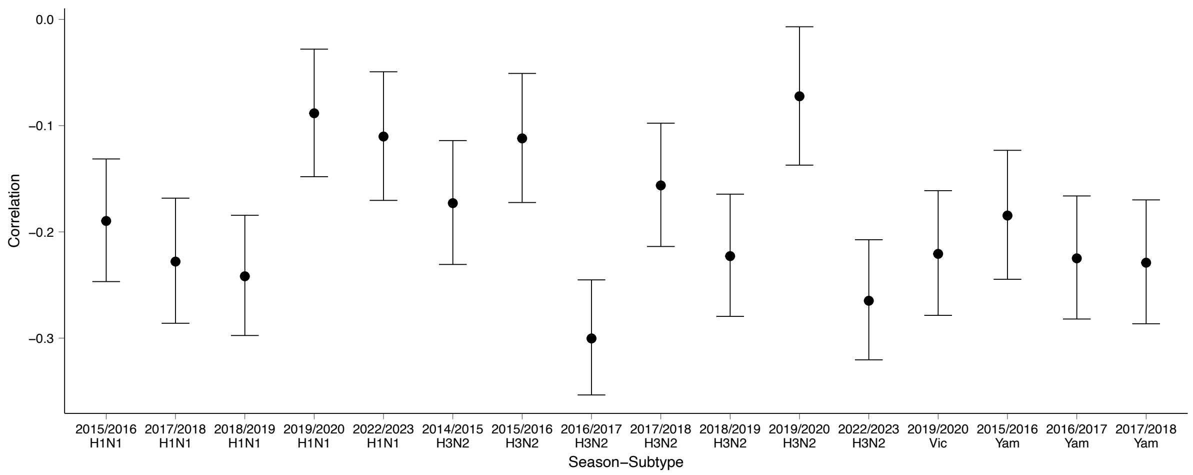

Supplementary Fig. 15. The Pearson correlation coefficient and associated 95% CI for the correlation between the log normalized pairwise jump frequency among states and log centroid distance, by subtype-season pair.
