# Supplementary material for: Competition between transmission lineages mediated by human mobility shapes seasonal influenza epidemics in the US
Source: Nat Commun. 2025 May 17;16:4605. doi: 10.1038/s41467-025-59757-4 (PMC12085627; doi:10.1038/s41467-025-59757-4)
Supplement: Supplementary file 3 — Description of Additional Supplementary Files [file 41467_2025_59757_MOESM3_ESM.pdf]

File Name: Supplementary Data 1
Description: GISAID Acknowledgement Table
