# Supplementary material for: Competition between transmission lineages mediated by human mobility shapes seasonal influenza epidemics in the US
Source: Nat Commun. 2025 May 17;16:4605. doi: 10.1038/s41467-025-59757-4 (PMC12085627; doi:10.1038/s41467-025-59757-4)
Supplement: Supplementary file 5 — Reporting Summary [file 41467_2025_59757_MOESM5_ESM.pdf]

Corresponding author(s): Simon de Jong

Last updated by author(s): 14/04/2025

Reporting Summary

Nature Portfolio wishes to improve the reproducibility of the work that we publish. This form provides structure for consistency and transparency in reporting. For further information on Nature Portfolio policies, see our Editorial Policies and the Editorial Policy Checklist.

Statistics

For all statistical analyses, confirm that the following items are present in the figure legend, table legend, main text, or Methods section.

n/a	Confirmed	
☐	☒	The exact sample size (*n*) for each experimental group/condition, given as a discrete number and unit of measurement
☐	☒	A statement on whether measurements were taken from distinct samples or whether the same sample was measured repeatedly
☐	☒	The statistical test(s) used AND whether they are one- or two-sided<br>*Only common tests should be described solely by name; describe more complex techniques in the Methods section.*
☐	☒	A description of all covariates tested
☒	☐	A description of any assumptions or corrections, such as tests of normality and adjustment for multiple comparisons
☐	☒	A full description of the statistical parameters including central tendency (e.g. means) or other basic estimates (e.g. regression coefficient) AND variation (e.g. standard deviation) or associated estimates of uncertainty (e.g. confidence intervals)
☐	☒	For null hypothesis testing, the test statistic (e.g. *F*, *t*, *r*) with confidence intervals, effect sizes, degrees of freedom and *P* value noted<br>*Give P values as exact values whenever suitable.*
☐	☒	For Bayesian analysis, information on the choice of priors and Markov chain Monte Carlo settings
☒	☐	For hierarchical and complex designs, identification of the appropriate level for tests and full reporting of outcomes
☐	☒	Estimates of effect sizes (e.g. Cohen's *d*, Pearson's *r*), indicating how they were calculated

Our web collection on statistics for biologists contains articles on many of the points above.

Software and code

Policy information about availability of computer code

Data collection	No software was used to collect the data in this study.
Data analysis	BEAST v1.10.5, TreeTime 0.8.1, IQTREE v.2.0.3, MAFFT v7.520 and CD-HIT v4.8.1 were used to perform the phylogenetic analyses. R packages MASS v7.3-54, vegan v2.6-2 were used to perform the analysis of lineage structure. Rcpp v.1.0.10 and RcppArmadillo v0.10.5.0.0 were used to interface with C++ for the epidemic simulations. R packages usmap v0.5.2 and ggtree v.3.2.1 were used for visualization. Analyses were performed with R v4.1.0. The procedure used to cluster the taxa is based on Phydelity 2.0. Computer code underlying the results of this paper is available at https://github.com/AMC-LAEB/usa_flu.

For manuscripts utilizing custom algorithms or software that are central to the research but not yet described in published literature, software must be made available to editors and reviewers. We strongly encourage code deposition in a community repository (e.g. GitHub). See the Nature Portfolio guidelines for submitting code & software for further information.

Data

Policy information about availability of data

All manuscripts must include a data availability statement. This statement should provide the following information, where applicable:

- Accession codes, unique identifiers, or web links for publicly available datasets
- A description of any restrictions on data availability
- For clinical datasets or third party data, please ensure that the statement adheres to our policy

Sequence data is available from GISAID. Epidemiological data is available from CDC FluView (https://gis.cdc.gov/grasp/fluview/fluportaldashboard.html). Commuting data is available from the US Census Bureau (https://www.census.gov/data/tables/2020/demo/metro-micro/commuting-flows-2020.html). Air transportation data is available from the US Department of Transportation (https://www.transtats.bts.gov/DL_SelectFields.aspx?gnoyr_VQ=GED&QO_fu146_anzr=).

Research involving human participants, their data, or biological material

Policy information about studies with human participants or human data. See also policy information about sex, gender (identity/presentation), and sexual orientation and race, ethnicity and racism.

Reporting on sex and gender	*Use the terms sex (biological attribute) and gender (shaped by social and cultural circumstances) carefully in order to avoid confusing both terms. Indicate if findings apply to only one sex or gender; describe whether sex and gender were considered in study design; whether sex and/or gender was determined based on self-reporting or assigned and methods used.*<br>*Provide in the source data disaggregated sex and gender data, where this information has been collected, and if consent has been obtained for sharing of individual-level data; provide overall numbers in this Reporting Summary. Please state if this information has not been collected.*<br>*Report sex- and gender-based analyses where performed, justify reasons for lack of sex- and gender-based analysis.*
Reporting on race, ethnicity, or other socially relevant groupings	*Please specify the socially constructed or socially relevant categorization variable(s) used in your manuscript and explain why they were used. Please note that such variables should not be used as proxies for other socially constructed/relevant variables (for example, race or ethnicity should not be used as a proxy for socioeconomic status).*<br>*Provide clear definitions of the relevant terms used, how they were provided (by the participants/respondents, the researchers, or third parties), and the method(s) used to classify people into the different categories (e.g. self-report, census or administrative data, social media data, etc.)*<br>*Please provide details about how you controlled for confounding variables in your analyses.*
Population characteristics	*Describe the covariate-relevant population characteristics of the human research participants (e.g. age, genotypic information, past and current diagnosis and treatment categories). If you filled out the behavioural & social sciences study design questions and have nothing to add here, write "See above."*
Recruitment	*Describe how participants were recruited. Outline any potential self-selection bias or other biases that may be present and how these are likely to impact results.*
Ethics oversight	*Identify the organization(s) that approved the study protocol.*

Note that full information on the approval of the study protocol must also be provided in the manuscript.

Field-specific reporting

Please select the one below that is the best fit for your research. If you are not sure, read the appropriate sections before making your selection.

☐ Life sciences ☐ Behavioural & social sciences ☒ Ecological, evolutionary & environmental sciences

For a reference copy of the document with all sections, see nature.com/documents/nr-reporting-summary-flat.pdf

Ecological, evolutionary & environmental sciences study design

All studies must disclose on these points even when the disclosure is negative.

Study description	This study is a retrospective analysis of seasonal influenza epidemic dynamics in the United States. Phylogenetic analyses were performed to characterize epidemic lineage structure. These analyses were integrated with epidemiological data to map and analyze the inter-state migration of viruses. These analyses are further supported by phylogeographic analyses to reconstruct migration pathways and analyze source-sink dynamics, and simulation analyses to determine the underlying determinants of lineage spread.
Research sample	For the genetic data, the research sample consisted of all seasonal influenza virus whole-genome sequences collected in the United States between 2014 and 2023, uploaded in the GISAID database with the US Centers of Disease Control as submitting laboratory and collected from humans. We restricted the analysis to the period from the 2014/2015 season as that was the first period with sufficient numbers of whole-genome sequences to afford state-level granularity in spread reconstructions.
Sampling strategy	For the analyses of lineage structure, we found a strong log-linear relationship between a state's population size and its sequencing

rate relative to its population size (Pearson r = -0.73, P < 0.001), but some states had substantially greater sampling rates than would be expected under the identified relationship given their population size. To minimize effects of differences in sampling on cluster delineations, we subsampled the taxa in each state, for each season-subtype pair, such that no state had a number of sequences more than 0.5 log units greater than the regression-predicted number given its population size.

For the phylogeographic analyses at the HHS region level, we used two different sampling strategies to account for and assess the effect of sampling heterogeneity on results. For our first sampling strategy, we used a sampling strategy where sequences from states that had a sequence count that was greater than expected from the regression line relating sequencing rate to population size were subsampled to the sequence count predicted from the regression line given its population size. This subsampling strategy was akin to the subsampling strategy used for the cluster delineations described above, but more stringent. Hence, the sample count for each HHS region was roughly proportional to the region's population size. For the second sampling strategy, we ensured that the number of taxa included for each HHS region was approximately uniform, irrespective of the HHS region's population size. For each season-subtype combination, we computed the sequence count as the 25th quantile of the number of sequences in each HHS region in the population-proportional subsampling scheme used above. For regions with more sequences than this value, sequences were randomly subsampled. Because the results of the inferences are subject to variation due to the sampling strategy used, we mainly reported among-state differences for any given sampling strategy, and de-emphasized the absolute proportions estimated using the different models. For the same reason, we reported the likely origins of the largest lineages averaged across both sampling strategies.

Data collection

Data was collected by downloading from online databases.

Timing and spatial scale

Not relevant as the study is an analysis of existing data.

Data exclusions

The states Alaska and Hawaii were excluded from the analyses as their epidemiological dynamics do not follow the typical temperate epidemiological dynamics of seasonal influenza viruses (Hawaii) and because the salient dynamics of mobility such as commuting are divergent between the conterminous states and Alaska and Hawaii. The 2020/2021 and 2021/2022 seasons were excluded from the analyses, as the atypical dynamics seen in these seasons (relative absence in 2020/2021, atypical circulation patterns and likely strong biases in the syndromic and virological data due to co-circulation of SARS-CoV-2 in 2021/2022, leading to unreliable estimates of weekly incidence across temporal and geographical units) preclude the accurate estimation of lineage size and onset timing in different states.

Reproducibility

This study does not consist of experimental work.

Randomization

Randomization is not relevant to the study as the study did not consist of experimental groups.

Blinding

Blinding is not relevant to the study as the study did not consist of experimental groups.

Did the study involve field work? ☐ Yes ☒ No

Reporting for specific materials, systems and methods

We require information from authors about some types of materials, experimental systems and methods used in many studies. Here, indicate whether each material, system or method listed is relevant to your study. If you are not sure if a list item applies to your research, read the appropriate section before selecting a response.

Materials & experimental systems

n/a	Involved in the study
☒	☐ Antibodies
☒	☐ Eukaryotic cell lines
☒	☐ Palaeontology and archaeology
☒	☐ Animals and other organisms
☒	☐ Clinical data
☒	☐ Dual use research of concern
☒	☐ Plants

Methods

n/a	Involved in the study
☒	☐ ChIP-seq
☒	☐ Flow cytometry
☒	☐ MRI-based neuroimaging

Plants

Seed stocks	*Report on the source of all seed stocks or other plant material used. If applicable, state the seed stock centre and catalogue number. If plant specimens were collected from the field, describe the collection location, date and sampling procedures.*
Novel plant genotypes	*Describe the methods by which all novel plant genotypes were produced. This includes those generated by transgenic approaches, gene editing, chemical/radiation-based mutagenesis and hybridization. For transgenic lines, describe the transformation method, the number of independent lines analyzed and the generation upon which experiments were performed. For gene-edited lines, describe the editor used, the endogenous sequence targeted for editing, the targeting guide RNA sequence (if applicable) and how the editor was applied.*
Authentication	*Describe any authentication procedures for each seed stock used or novel genotype generated. Describe any experiments used to assess the effect of a mutation and, where applicable, how potential secondary effects (e.g. second site T-DNA insertions, mosiacism, off-target gene editing) were examined.*
